# Projected changes in distributions of Australian tropical savanna birds under climate change using three dispersal scenarios

**DOI:** 10.1002/ece3.197

**Published:** 2012-04

**Authors:** April E Reside, Jeremy VanDerWal, Alex S Kutt

**Affiliations:** 1 CSIRO Climate Adaptation Flagship and Ecosystem SciencesPrivate Mail Bag PO, Aitkenvale, Queensland, 4814, Australia; 2 Centre for Tropical Biodiversity and Climate Change, School of Marine and Tropical BiologyJames Cook University, Townsville, Queensland, 4811, Australia

**Keywords:** Conservation, dispersal, migration, Maxent, niche, species distribution modelling, vulnerability

## Abstract

Identifying the species most vulnerable to extinction as a result of climate change is a necessary first step in mitigating biodiversity decline. Species distribution modeling (SDM) is a commonly used tool to assess potential climate change impacts on distributions of species. We use SDMs to predict geographic ranges for 243 birds of Australian tropical savannas, and to project changes in species richness and ranges under a future climate scenario between 1990 and 2080. Realistic predictions require recognition of the variability in species capacity to track climatically suitable environments. Here we assess the effect of dispersal on model results by using three approaches: full dispersal, no dispersal and a partial-dispersal scenario permitting species to track climate change at a rate of 30 km per decade. As expected, the projected distributions and richness patterns are highly sensitive to the dispersal scenario. Projected future range sizes decreased for 66% of species if full dispersal was assumed, but for 89% of species when no dispersal was assumed. However, realistic future predictions should not assume a single dispersal scenario for all species and as such, we assigned each species to the most appropriate dispersal category based on individual mobility and habitat specificity; this permitted the best estimates of where species will be in the future. Under this “realistic” dispersal scenario, projected ranges sizes decreased for 67% of species but showed that migratory and tropical-endemic birds are predicted to benefit from climate change with increasing distributional area. Richness hotspots of tropical savanna birds are expected to move, increasing in southern savannas and southward along the east coast of Australia, but decreasing in the arid zone. Understanding the complexity of effects of climate change on species’ range sizes by incorporating dispersal capacities is a crucial step toward developing adaptation policies for the conservation of vulnerable species.

## Introduction

Global climate change is already having an effect on species and communities, with severe impacts expected across taxonomic groups with increasingly rapid climate change (Walther et al. 2002; Thomas et al. 2004). Climate change has resulted in species’ Grinnellian niche—defined as the environmental conditions characterizing its occurrence (Grinnell 1917)—shifting to new geographic locations (Tingley et al. 2009). The Grinnellian niche is well represented by climate for many species, and is commonly referred to as its suitable climate space (Root 1988; Kearney and Porter 2009). Many species have been documented as tracking the shifts in their suitable climate space to new geographic locations (Tingley et al. 2009) and generally this shift is toward the poles or higher altitudes as temperatures increase (Parmesan and Yohe 2003). However, rising temperatures combined with changing precipitation patterns can have more complex effects on species distributional shifts, and some species’ suitable climate spaces are projected to disappear altogether (Williams et al. 2003; Malcolm et al. 2006; Williams and Middleton 2008; Coetzee et al. 2009). The increased extinction risk predicted for many species due to climate change has resulted in conservation initiatives to incorporate vulnerability to climate change as a factor for listing a species as threatened and requiring management intervention (Brook et al. 2009; Hawke 2009). With up to 50% of the world's biodiversity already threatened with extinction (Millennium Ecosystem Assessment 2005) and biodiversity continuing to decline (Secretariat of the Convention on Biological Diversity 2010), broad-scale assessments of species’ vulnerabilities to climate change are needed to prioritize those in need of urgent conservation action.

Tropical biota are expected to have higher than average sensitivity to climate change, due to the high species diversity, smaller ranges, and narrower thermal tolerances (Colwell et al. 2008; Deutsch et al. 2008). Species restricted to high altitude tropical regions face “mountain top extinctions” as their suitable climate space shifts upslope with rising temperatures (Williams et al. 2003). Tropical lowlands are predicted to decrease in species richness, as there are no species currently in hotter places available to replace those that move to higher latitudes or altitudes (Colwell et al. 2008). In Australia, modelling studies on climate change impacts on tropical rainforest fauna forecast severe declines in the area of suitable climate space and possible species extinctions, particularly for species confined to tropical uplands (Williams et al. 2003; Hilbert et al. 2004; Shoo 2005). However, over 60% of tropical Australia is savanna and few assessments of the consequences of climate change for species occupying this biome have been conducted. One example examining kangaroo (Macropodidae) distribution in northern Australia predicted average range reductions per species of 48% with 2°C of warming (Ritchie and Bolitho 2008). A more in-depth understanding of the climate change threat to species persistence in Australian tropical savannas is imperative, as many vertebrate populations are declining at sufficient rates to be cause for concern. In particular, small mammals (Woinarski et al. 2010, 2011) and granivorous birds (Franklin et al. 2005) are declining as a result of altered habitat conditions due to changed fire regimes and widespread cattle grazing, despite most of the region being relatively unmodified (Fensham et al. 1999; Russell–Smith 2002).

Many studies have documented mobile species such as birds shifting their ranges and migration strategies in response to change in climate and weather patterns (Dunlop and Wooller 1986; Pounds et al. 1999; Thomas and Lennon 1999; Cotton 2003; Reid 2003; Brommer 2004; Austin and Rehfisch 2005; Beaumont et al. 2006; Visser et al. 2009). These responses are likely to amplify as global climates continue to change in line with projections (IPCC 2007b). Birds are an exemplar study group for understanding and anticipating the potential effect of climate change because more is known about their distributions and life histories than many other taxa (Webb and Gaston 2000). In Australia, range shifts of birds have been documented in recent years; but attributing this observation to climate change is confounded by the relative effects of land-use change on bird movements and distributions (Chambers et al. 2005). Despite this complexity, there is evidence for climate change contributing to species declines in southern Australia (Mac Nally et al. 2009), Western Australia (Rowley and Russell 2002), and for seabird populations in the northeast (Smithers et al. 2003).

Birds of the Australian tropical savanna biome have complex and flexible movement patterns and therefore highly adapted to variable resource distributions (Chan 2001; Woinarski et al. 2000). Despite the general mobility of savanna bird species, some are habitat or food specialists with restricted distributions (Weaver 1982; Rowley and Russell 1993; Perry et al. 2011). While some species have the adaptive capacity to track suitable climate space as it shifts geographically, some species may be constrained by the time required for habitats (e.g., vegetation) to change in response to changing climate (Warren et al. 2001). Therefore, forecasting actual climate change responses by individual species requires realistic dispersal scenarios. Ideally, the dispersal scenario should be tailored to be as accurate for individual species as current knowledge will allow, in order to account for variation in individual species dispersal ability (le Roux and McGeoch 2008). These realistic dispersal scenarios improve projections by predicting not only the direction in which species’ suitable climate spaces are shifting, but also the ability of species to track the shift, including accounting for species’ habitat limitations (Midgley et al. 2006). Generally species are expected to move to higher latitudes (Parmesan and Yohe 2003). For birds of the Australian tropical savanna, direct poleward movement of many species may be impeded by the arid zone on the southern boundary of the biome, and the disjunction between wooded savanna and largely treeless grassland and desert (Mott et al. 1985). While global studies on future climate suggest that while the broad climatic biome classification of northern Australia are unlikely to shift substantially (Rubel and Kottek 2010), this region is expected to experience climates that are relatively novel (Williams et al. 2007). However, it is unknown how the suitable climate space of savanna birds will change on a regional scale, how well different species will be able to track that movement, and as a consequence, what will happen to the species richness of the tropical savannas. In this study, we investigate the impact of future climate change on the bird fauna of Australian tropical savannas. We use distribution models for 243 species to: (1) estimate the change in species richness between 1990 and 2080; (2) investigate the effects of different dispersal scenarios on species potential response to climate change; and (3) using a realistic dispersal scenario for each species, estimate the potential impact of climate change on individual species, and across groupings of: (a) autecology and (b) current conservation concern under Australian and international listings.

## Materials and Methods

### Study area

The Australian tropical savannas occur north of ca. 23^o^S (Franklin et al. 2005), occupying nearly one-quarter of the continent (Williams et al. 2005). Savannas are characterized by a discontinuous stratum of trees above a mostly continuous layer of grasses (Williams et al. 2002; Lehmann et al. 2011). Rainfall is highly seasonal and largely occurs in the wet season between December and March (Felderhof and Gillieson 2006). A climatic gradient extends from the higher rainfall savannas near the coast, to the semi-arid savannas inland with increased interannual rainfall variability (Mott et al. 1985).

### Bird data

We focused our study on 243 bird species occurring within the northern tropical savanna woodlands excluding waterbirds and rainforest species that may occur intermittently in savanna regions. Bird occurrence records were collated from the Birds Australia Atlas (Blakers et al. 1984; Barrett et al. 2003), the Queensland Governmental atlas WildNet (Environmental Protection Agency 2004), and CSIRO (protocol as in Reside et al. 2010). The mean number of records per species was 23,027 (range: 6–34,330), and the occurrence records spanned from 1950 until 2009. Species were grouped according to their movement life history (migratory, nomadic, sedentary, partially migratory, and species that were both nomadic and sedentary). Most species that occur within Australian tropical savannas also occur beyond the savanna region, many occurring widely across Australia. The species were grouped into five broad biogeographic groups describing their broader range: arid, Cape York Peninsula, temperate, tropical, and ubiquitous for species that encompassed two or more of the above categories; according to the literature (Schodde 1981; Marchant and Higgins 1990; Marchant and Higgins 1993; Higgins and Davies 1996; Higgins 1999; Higgins et al. 2001; Higgins and Peter 2002; Higgins et al. 2006). Details for each species are provided in the Supporting information. While we focused our study on the suite of species that occur in the tropical savannas, we investigated the effect of climate change on species’ broader ranges, even when they extend beyond the savanna and across the rest of Australia. Detailed explanations of the biogeographic groupings can be found in Reside et al. (2010). Species conservation status was also compiled. Nineteen of 243 species in our study are listed as having a significant conservation status under the Australian Commonwealth Government (EPBC: Environment Protection and Biodiversity Conservation Act 1999), Queensland Sate (NCA: Nature Conservation Act 1992), or international (IUCN 2001) categories of endangered, vulnerable or near-threatened (Table 2).

**Table 1:** The eight global circulation models used for the projections of future climate. The number of runs for the 20th (C20) and 21st (C21) century, and the total number of realizations, used for the future projections are shown for each GCM (Cubash et al. 2001).

Global circulation model	No. of runs for C20	No. of runs for C21	No. of realizations
BCCR-BCM 2.0	1	1	1
CSIRO-Mk 3.0	3	1	3
CSIRO-Mk 3.5	3	1	3
GISS AOM	2	2	4
INM CM 3.0	1	1	1
MIROC 3.2 (hires)	1	1	1
MIROC 3.2 (medres)	3	3	9
NCAR CCSM 3.0	2	4	8

### Climate data

The climate data used for modelling were grided spatial layers of annual mean temperature, temperature seasonality (the standard deviation of the weekly mean temperatures expressed as a percentage of the annual mean), maximum temperature of the warmest period, annual precipitation, precipitation seasonality, and precipitation of the driest period. These variables have been shown to produce robust species distribution models for vertebrates in northern Australia (VanDerWal et al. 2009a, b; Williams et al. 2009; Reside et al. 2010). The climate layers were derived from monthly climate surfaces obtained from the Australian Water Availability Project (Jones et al. 2007; Grant et al. 2008) averaged over the period 1961–1990 at a 0.05^o^ resolution (∼5 km grid). The climate layers were created using the “climates” package in *R* (VanDerWal et al. 2011), and are equivalent to the bioclim variables derived using Anuclim 5.1 software (Hutchinson et al. 2000).

### Climate projections

**Figure 1:**
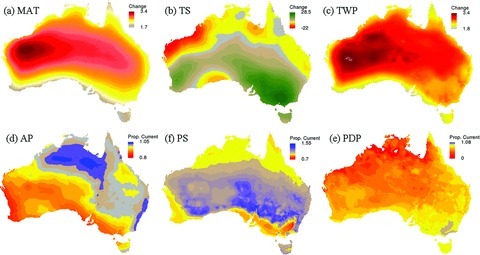
The change in climate between mean projections for 1990 and 2080, modeled at a 0.05^o^ resolution. Thirty climate projections representing eight different global circulation models (GCMs) and multiple realizations for each GCM were summarized first within GCM and then across GCMs to give the mean projection for each year. The climate variables used are mean annual temperature (MAT), temperature seasonality (TS), temperature of the warmest period (TWP), annual precipitation (AP), precipitation seasonality (PS), and precipitation of the driest period (PDP). The scale bars show the absolute change in temperate variables between the 1990 baseline and the 2080 projection for (A–C); and (D and E) the proportional change between 1990 and 2080 for rainfall variables. The units for the temperature variables are degrees Celsius. Higher values for seasonality correspond with increasing seasonality.

Climate projection layers consisted of climate surfaces for 10-year intervals between 1990 and 2080. The layers used for each of the 10-year intervals were a 30-year average around that year; for example, the climate representing “1990” was climate averaged from 1985 to 2005, the climate representing “2000” was the climate averaged from 1995 to 2015, etc. The future climate surfaces were based on the IPCC Special Report on Emission Scenarios (SRES) scenario A1B, which represents a medium-severity projection of both fossil fuel and nonfossil fuel energy sources (Nakicenovic et al. 2000). Future climate surfaces were based on eight global circulation models (GCM) (Cubash et al. 2001) as used in the IPCC Fourth Assessment Report (IPCC 2007a), some with multiple realizations (Table 1), resulting in 30 projections per 10-year interval. Figure 1 presents the weighted mean change in each climate variable between 1990 and 2080. The weighting was based on the number of realizations per GCM to remove any possible GCM-specific bias. By 2080, both mean annual temperature and temperature of the warmest period are projected to increase the most in the Pilbara and Great Sandy Desert bioregions of northwestern Australia, by up to 3.4°C (Fig. 1). The increase in temperature declines with decreasing distance to the coast. This is in broad agreement with other work projecting future climate in Australia (Whetton et al. 2005). Temperature seasonality is projected to decrease in northern Australia (less variation throughout the year in temperature) and increase across the south.

**Table 2:** The species in our study listed as critically endangered (CE), endangered (E), vulnerable (V), or near-threatened (NT) under the federal (EPBC: Environment Protection and Biodiversity Conservation Act 1999), state (NCA: Nature Conservation Act 1992), or international (IUCN 2001) classifications, including those with threatened subspecies (subsp.). The “Proportion of current” column gives the proportional change that the future range is projected to be in 2080 in relation to the current range size.

Species	EPBC	NCA	IUCN	Proportion of current	Direction of change in area
Buff-breasted button quail *Turnix olivii*	E	V	E	0.17	Decrease
Golden-shouldered parrot *Psephotus chrysopterygius*	E	E	E	0.06	Decrease
Gouldian finch *Erythrura gouldiae*	E	E	E	1.76	Increase
Red goshawk *Erythrotriorchis radiatus*	V	E	V	1.32	Increase
Painted honeyeater *Grantiella picta*		V		0.62	Decrease
Purple-crowned fairy-wren *Malurus coronatus*	V subsp	V		1	No change
Yellow chat *Epthianura crocea*	CE subsp	V		1.62	Increase
Crimson finch *Epthianura tricolor*	V subsp	V		1.55	Increase
Grey goshawk *Accipiter novaehollandiae*		NT		1.33	Increase
Grey falcon *Falco hypoleucos*		NT	NT	0.89	Decrease
Square-tailed kite *Lophoictinia isura*		NT		1.07	Increase
Palm cockatoo *Probosciger aterrimus*		NT		0.05	Decrease
Pictorella mannikin *Heteromunia pectoralis*		NT		1.45	Increase
Australian Bustard *Ardeotis australis*			NT	1.24	Increase
Bush stone curlew *Burhinus grallarius*			NT	1.14	Increase
Squatter pigeon *Geophaps scripta*	V subsp	V subsp	V subsp	0.71	Decrease
Double-eyed fig-parrot *Cyclopsitta diophthalma*	E subsp	E, V, NT subsp		0.80	Decrease
Black-throated finch *Poephila cincta*		E subsp	E subsp	0.40	Decrease
Star finch *Neochmia ruficauda*	E subsp	E subsp		1.24	Increase

The climate projections indicate that precipitation may increase across the central tropical savanna by up to 5%; but decrease across northwestern Australia and southern Australia. Precipitation seasonality is projected to decrease slightly in northern savannas, and precipitation of the driest period is predicted to decrease across most of the continent. The extreme changes (maximum and minimum, Appendix S1) show similar spatial patterns in increases in annual mean temperature between 1990 and 2080, with varying degrees of warming. Changes in annual precipitation are more varied: the minimum shows the whole continent getting drier, with large tracts of inland Australia receiving half the current annual rainfall; whereas the maximum shows most of the continent receiving more rainfall by up to 184%.

### Distribution models

Species distribution models incorporating baseline climate data and species occurrences were created using Maxent (Phillips et al. 2006). Maxent uses presence-only data to statistically relate distribution records to environmental variables on the principle of maximum entropy. Default settings were used as these have been optimized for numerous species across many regions (Phillips and Dudik 2008). Models were run at a spatial resolution of 0.05° (ca. 5 × 5 km). We used the default of 10,000 background points sampled from across Australia, which for most species here is the most appropriate background, as many of the species modeled are widely distributed across the continent (VanDerWal et al. 2009a). We acknowledge that for the smaller ranged species, this background may provide a liberal estimate of the distributions; however, we wanted to use a consistent background for all species. Model performance was evaluated by the area under the receiver operating characteristic curve (AUC). AUC measures each models’ consistency and predictive accuracy (Ling et al. 2003). An AUC score of 1 is a perfect model fit of the data; 0.5 is no better than random (Elith et al. 2006, Phillips et al. 2006). AUC values ≥ 0.7 indicate “useful” models, while values ≥ 0.9 indicate models with “high” performance (Swets 1988). Models for each species were screened for low AUC (<0.7) so that underperforming models were not included in further analyses. Model performance was generally high: AUC scores ranged from 0.66 to 0.99, with 82 species having AUC scores greater than 0.95. Eleven species had AUC scores less than 0.7 and so were excluded from species-specific analyses (see Supporting information).

### Species’ range projections

Species models were projected onto each of the 300 future climate surfaces (30 realizations across 8 GCMs per decade). These were averaged to examine the weighted mean and extremes as an ensemble model. The mean was weighted by the number of realizations per GCM to avoid undue influence by GCMs with more realizations; and the extremes were calculated as the minimum and maximum projections. The projections represent “potential” future distribution ranges, which are the suitable climate space based on the current Grinnellian niche for the species. Potential distributions are often an overestimate of species actual, “realized” distribution (Anderson et al. 2003); therefore realized distributions were created by clipping the current potential distribution to the subregions for which the species has, at some time, been observed. The subregion boundaries are ecologically relevant biogeographic regions defined by the Interim Biogeographic Rationalization for Australia, Version 6.1 (Environment Australia 2000; Williams et al. 2010). Although there is a sampling bias for birds toward populated areas, there was sufficient sampling across bioregions such that we could realistically suggest that if a species has never been reported in a bioregion, the region was treated as unsuitable for the species beyond climatic suitability (e.g., dispersal limitations, unsuitable vegetation, and competition). For example, it is likely a dispersal limitation preventing a species confined to east coast of Australia from occurring in suitable climate on the west coast due to the 3000 km of unsuitable matrix separating the environments. The future species’ range projections were limited to three dispersal scenarios: full dispersal (no clipping), a realistic dispersal scenario of 3 km per year (applied as 30 km per decade), and no dispersal (i.e., species were constrained to the subbioregions that they currently occur in). Different studies documenting range shifts of birds have found that, averaged across the assemblage for each study, birds can shift their ranges from between 100 m to 5 km per year (Thomas and Lennon 1999; Brommer 2004; Devictor et al. 2008; Tingley et al. 2009; Zuckerberg et al. 2009; Martinez–Morales et al. 2010). We chose 3 km per year as an intermediate of these observed dispersal distances. The 3 km per year dispersal scenario represented the intersection of the future potential distribution (full-dispersal scenario) with the current realized distribution buffered by 3 km × number of years into the future being examined. Thus, for each 10-year period from 1990 to 2080, the current distribution was buffered (extended) by 30 km, resulting in 10 dispersal masks for each species. Each species was assigned to one of the three dispersal categories (full, 3 km per year, or no dispersal) as a best estimate of likelihood of dispersal ability, herein referred to as “realistic” dispersal. This estimate was based on the long-distance movements recorded in the literature, and by the current habitat specificity of the species (Marchant and Higgins 1990, 1993; Higgins and Davies 1996; Higgins 1999; Higgins et al. 2001, 2006; Higgins and Peter 2002). Species with greater habitat specificity were assumed to be less able to establish a new range without corresponding shifts in their preferred habitat, while species with generalist habitat associations are more likely to be able to track their climatic niche as it shifts (Warren et al. 2001). Estimates of realistic dispersal from the literature were corroborated with expert opinion (Eric Vanderduys pers. comm.), resulting in 197 species in the “full dispersal,” 28 species in the “3 km per year,” and seven species in the “no dispersal” categories, respectively.

The default Maxent distribution output is a continuous prediction of environmental suitability for the species. A binary distribution output was created by applying an appropriate threshold obtained from the Maxent results output file. The threshold showing the most realistic distributions for the species was the “equate entropy of threshold and original distributions logistic threshold.” All areas for the distribution of each species that the probability of presence fell below this species-specific threshold were accorded a “0,” and all areas equal to and greater than this threshold were accorded “1” or presence. The details of the threshold value for each species are given in the Supporting information.

Summary characteristics of each species projected distribution range, such as the total area, number of patches, proportion of the landscape, and statistics related to fragmentation were calculated using the “ClassStat” function of the SDMTools package from the CRAN website http://cran.r-project.org/web/packages/SDMTools/. Species richness maps were created by stacking all the binary distribution outputs for each species for each 10-year interval. The species richness maps included all 243 species to achieve more realistic species richness estimates, including species with low AUC scores as accuracy of individual species models was not vital given the of the large scale of the output. All analyses were conducted using the statistical package “R” version 2.12.1 (http://www.r-project.org).

## Results

### Species richness

**Figure 2:**
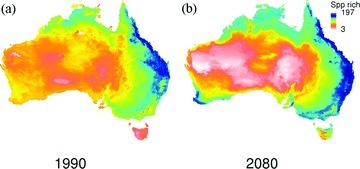
Species richness of savanna bird species, calculated by stacking each species’ Maxent model for 1990 (A), compared to the species richness projected for 2080 (B).

Species richness of the savanna bird assemblage is projected to change across the savanna region and across Australia with some notable shifts projected between 1990 and 2080 for the realistic dispersal scenario (Fig. 2). Savanna bird species richness is projected to decrease in the arid zone, particularly in western regions. This contrasts with the increase in species richness projected for the southern savannas, and eastward and southward along the east coast of the continent by 2080 (Fig. 2B). Projected increases in species richness correspond with projected increases in annual precipitation within the savannas (Fig. 1D). Savanna regions in which a decrease in species richness is projected are those likely to experience the greatest increase in temperatures, both annually (MAT) and during the humid summer (TWP) (Fig. 1A and 1C).

**Figure 3:**
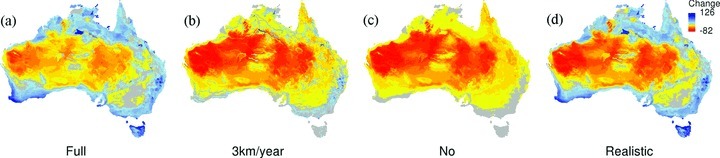
The change in species richness of savanna bird species between 1990 and 2080 depending on dispersal scenario. “Full” is unlimited dispersal (A), “3 km/year” is dispersal at a rate of 3km per year (B), “No” is no dispersal (C), and “Realistic” (D) the best estimate of dispersal ability for each species. Warm colors indicate a reduction in species richness (yellow is the least lost and red the most); cool colors indicate an increase in species richness.

The extent of potential decreases and increases in species richness of savanna birds varies greatly depending on the dispersal scenario (Fig. 3). Assuming full dispersal is possible for all species; most of the tropical savannas are projected to increase in species richness (Fig. 3A). Under the full dispersal scenario, most of the decreases in species richness are confined to the arid zone. Increases in species richness of savanna species are projected for most of eastern Australia, Tasmania and southwest Western Australia. Few areas are projected to increase in species richness if all species are restricted to a dispersal rate of 3 km per year (Fig. 3B). The arid interior of the Australian continent remains the region of greatest potential loss of savanna species, with some small increases in species richness throughout the savanna and southward along the east coast. If no dispersal occurs, all of Australia will decrease in savanna species (Fig. 3C). The near-coastal northern savanna and southeastern Australia will face the least decrease in savanna species richness. Under restricted or no-dispersal scenarios, species will be unable to move to similar climate-niche areas of southwestern Australia, and southeastern Australia including Tasmania (Fig. 3B and 3C). Our realistic dispersal scenario shows species richness changes somewhat intermediate between the full dispersal and the 3 km per year dispersal scenarios (Fig. 3D). The reduction in species richness of savanna birds in the arid zone is greater for the realistic dispersal scenario compared with full dispersal, but many regions are projected to show increases in species richness.

### Projected changes for species

**Figure 4:**
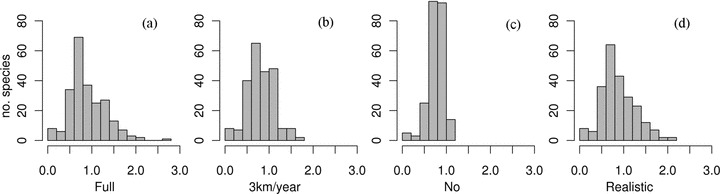
Histograms representing the proportional change in species’ suitable climate area between 1990 and 2080 according to the full (A), 3 km per year (B), no (C), and realistic (D) dispersal scenarios. The scale on the x-axis represents the proportional change, with 1 representing no change, 0 representing a complete loss of suitable climate space, and 3 representing a tripling of suitable climate space.

The proportional change in individual species distributions between 1990 and 2080 varied greatly depending on which dispersal scenario was used (Fig. 4). Under a full dispersal scenario, 154 species are expected to experience a decrease in their suitable climate space (Fig. 4A). Of the 78 distributions projected to increase, the average increase is 35% and the greatest increase is 164%. The number of species projected to increase in suitable climatic space is reduced to 66 species with an average of 16% with dispersal limited to 3 km per year (Fig. 4B). By definition, no increase in distribution is possible under a no-dispersal scenario (Fig. 4C). With a 3-km dispersal scenario, the suitable climate spaces for 166 species are projected to decrease, and with no dispersal this increases to 207 species.

Under the realistic dispersal scenario, the overall mean area percentage shift in suitable climate space for species is –13%. Decreases are projected for 155 (ca. 67%) bird species by 2080, by an average of 34%. One-third of species are projected to increase their area of suitable climate space by 2080. The average projected increase is 30%, and 16 species are likely to increase by 50%. Despite the large proportion of declines and the number of severe declines, 47 (20%) species are projected to change very little by 2080, only increasing or decreasing the size of their suitable climate space by less than 10%. From here on, all results will be discussed in terms of the realistic dispersal scenario for each species. Details of the proportional change in suitable climate space for each species are provided in the Supporting information.

### Autecology and biogeography for shifting species

**Figure 5:**
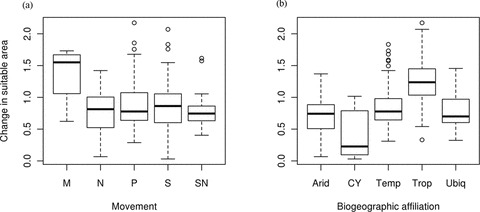
Boxplots showing the proportional change in species climatic niche area in relation to species movement life history and biogeographic affiliation, using a realistic dispersal scenario. Movement categories are migratory (M), nomadic (N), partially migratory (P), sedentary (S), and both sedentary and nomadic (SN). Biogeographic affiliation categories are arid, Cape York (CY), temperate (Temp), tropical (Trop), and ubiquitous (Ubiq).

**Figure 6:**
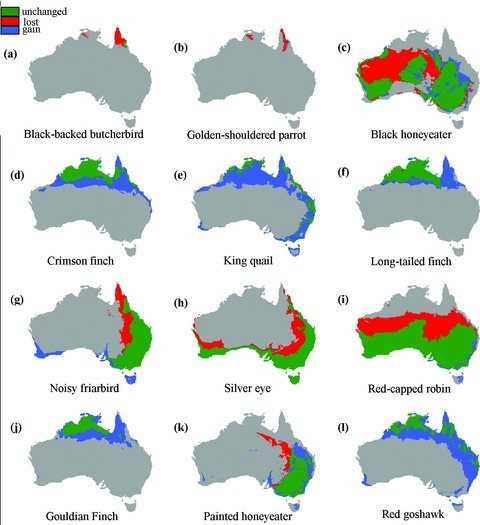
Examples of current and future predicted distributions showing some of the patterns found among tropical savanna birds using a realistic dispersal scenario. The green color indicates area that is suitable both in 1990 and 2080, red indicates area that was suitable in 1990 but not in 2080, and blue areas are gained by 2080. Examples shown are: Cape York species predicted to have the greatest proportional decrease in climatic niche (A and B); (C) an arid species predicted to face a severe decline in range; (D–F) examples of tropical species predicted to increase in range. (G–I) Species predicted to lose the Cape York section of their range or the inland margin. Some of the changes to threatened species climatic niche changes are shown in (J–L).

Migratory species are projected to have the greatest distribution increases, with no difference between other movement categories (Fig. 5A, *F*= 2.73, *P*= 0.03). Species with a “tropical” biogeographic affiliation showed on average the greatest increases in distribution, while “Cape York Peninsula” (CYP) species decreased the most (Fig. 5B, *F*= 29.45, *P* < 0.001). Of the eight species projected to lose more than 80% of their suitable climate space, six are largely restricted to northern CYP (black-backed butcherbird *Cracticus mentalis*, Fig. 6A; palm cockatoo *Probosciger aterrimus*; golden-shouldered parrot *Psephotus chrysopterygius*, Fig. 6B; tawny-breasted honeyeater *Xanthotis flaviventer;* white-streaked honeyeater *Trichodere cockerelli* and buff-breasted button-quail *Turnix olivii*), and the remaining two (black honeyeater *Sugomel niger*, Fig. 6C, and crimson chat *Epthianura tricolor*) are distributed throughout arid Australia. The CYP species that are expected to experience decreases in their suitable climate space are projected to lose the western edge of their range; becoming restricted to the cooler upland “refugial” areas of the Eastern Cape (Fig. 6A and 6B). The CYP decreasers are unlikely to be able to extend their distributions directly south to adjacent regions, as these will face greater increases in temperature than the rate of change in their current distribution (Fig. 1). Many species distributed along the east coast and partly occurring on CYP are projected to lose the CYP part of their range in the same manner as the CYP restricted species; that is, the western edge of their range is eroded while the cooler upland suitable climate space is retained.

Many species occurring across the arid zone are projected to lose the part of their range that encompasses western Australia, around the Pilbara and Great Sandy Desert bioregions (approximately 21°00′S, 124°00′E). This region is expected to experience the greatest increase in mean temperatures (Fig. 1A and 1C), and declines in species richness projections (Fig. 2B). Many species with distributions currently extending down the east coast of Australia are projected to lose the inland edge of their range, presumably related to the higher temperature gradient.

### Currently threatened species

Of the nineteen species currently listed as threatened, eight are projected to increase in distribution by 2080, 10 are projected to decrease and one is likely not to change in distribution (Table 2). Three threatened species are within the group projected to decline on CYP (golden-shouldered parrot, Fig. 6B; buff-breasted button quail and palm cockatoo). Two species listed as endangered, Gouldian finch *Erythrura gouldiae* and red goshawk *Erythrotriorchis radiatus*, are projected to increase in suitable climate space (Fig. 6J and 6L).

## Discussion

This study has demonstrated that decreases in distribution are expected for over two-thirds of Australian tropical savanna birds by 2080 based on their suitable climate space. However it should be noted that all the projections for species shown here are based on the weighted mean of 30 climate realizations derived from eight different GCMs each with several realizations (Table 1), and therefore the actual manifestation of future climate could diverge from these mean projections. The projections used here are based on the SRES A1B emissions scenario, which is a conservative mid-range scenario. This contrasts with the current rate of increase in global CO_2_ emissions since 2000 that is greater than the most severe projection developed by the IPCC in the late 1990s (Raupach et al. 2007). As a consequence, our projections for birds of the Australian tropical savanna are conservative. There is a significant potential for faster and more extreme change in suitable climate space further reducing the ability of many species to track this change.

The choice of dispersal scenario affects the predicted change in species richness, which varies from continent-wide decreases to large areas of increasing species richness. Many studies include a no-dispersal scenario in their projections, which is likely to be unrealistic given the natural plasticity in the distribution of most birds (Webb and Gaston 2000; Jetz and Rahbek 2002; Coetzee et al. 2009; Marini et al. 2009). Bird species have been recorded shifting their ranges in the Northern Hemisphere (Thomas and Lennon 1999; Brommer 2004; Tingley et al. 2009; Zuckerberg et al. 2009; Martinez–Morales et al. 2010), though these range shifts have lagged behind the spatial shifts of climate (Devictor et al. 2008).

For this reason, we chose an intermediate dispersal scenario to simulate a realistic projection into future locations of species ranges. In this study, the species richness projection for 2080 based on realistic dispersal scenarios for each species is most similar to the full dispersal projection, although declines in the arid zone are similar to those projected for the 3 km per year dispersal scenario (Fig. 3B).

The projected increases in species richness of savanna birds extend across most of the tropical savanna region under a realistic dispersal scenario. This is true for the coastal lowlands and the mid-elevational regions, coinciding with the projected increases in rainfall in the region. In contrast to the prediction that tropical lowlands are likely to lose the most species (Colwell et al. 2008), our study projects that it is the higher elevation areas within the region that are predicted to face a reduction in species richness. However, our study only looks at projections of tropical savanna bird species, therefore the actual bird species richness of the region may differ due to different responses by birds that are currently restricted to rainforest or arid areas.

Tropical savanna bird species that migrate annually north beyond the Australian continent (e.g., eastern koel *Eudynamys orientalis*, oriental cuckoo *Cuculus saturatus* and dollarbird *Eurystomus orientalis*) are projected to benefit the most from climate change. These species are expected to extend their range down the east coast and into areas in which rainfall is projected to increase; a response to climate change that may already be occurring (Reid 2003). Aside from assigning each species to a dispersal scenario, our modeled projections of future range are based on the bioclimatic correlates of current distribution for each species and do not take behavior into account. However, migratory behavior is likely to enhance species’ adaptive capacity in response to climate change; as migratory species already disperse to suitable habitat with changing weather patterns (Şekercioğlu 2007).

In general, species distributed predominantly across northern Australia, the “tropical” distribution (e.g., Fig. 6D, 6F, and 6J), are projected to fare the best with future range expansion south and east in tandem with increasing rainfall. In contrast, large range decreases are projected for the narrow-ranged species currently found on Cape York Peninsula. These species are likely to be the most vulnerable to extinction. This fits with the theory that diverse tropical assemblages consisting of small-ranged species have the highest vulnerability to climate change (Colwell et al. 2008). These species may be on the edge of their thermal tolerances, as they occupy one of the hottest regions in the continent (Deutsch et al. 2008). The western side of the Cape currently has higher annual mean temperature and lower dry-season precipitation than the eastern side, and these western regions are projected to become unsuitable (Fig. S1). For the three species on Cape York Peninsula projected to face severe declines that are currently listed as threatened (golden-shouldered parrot, buff-breasted button quail and palm cockatoo), the combination of climate change and their current threatening processes (e.g., inappropriate fire regimes and grazing; Garnett and Crowley 2002; Mathieson and Smith 2009) is likely to lead to a high risk of extinction. For those that rely on specific nesting requirements, for example, termite mounds for golden-shouldered parrot and hollow-bearing trees for palm cockatoo, their vulnerability is exacerbated by the risk that climate change will interrupt the crucial biotic interactions they depend upon through changes in fire or cyclonic activity (Weaver 1982; Murphy and Legge 2007).

These projected species-specific responses are likely to result in substantial changes in species composition across the Australian tropical savannas and the rest of Australia. Migratory and tropical species are likely to become more widespread while species inhabiting the savannas at the southern edge (e.g., arid-affiliated species) are likely to be lost from the savanna region. Northern Australia may receive more migrants from Papua New Guinea and southeast Asia, which may expand their ranges south. Potential changes could result in “no-analogue” species assemblages due to community reorganization (le Roux and McGeoch 2008). Compositional changes in bird species assemblages have already occurred in response to climatic change in other regions (Albright et al. 2010). In particular, generalist species have increased while specialists decreased (Christian et al. 2009). This has been shown for butterfly populations, where increases in species richness lag behind the predicted increases, with the resultant species assemblages showing a greater dominance of generalist species (Warren et al. 2001; Menéndez et al. 2006). In Australia, widespread generalist bird species, such as crested pigeon (*Ocyphaps lophotes*) and galah (*Eolophus roseicapillus*), have increased their ranges across Australia largely as a result of land-use change (Franklin 1999); generalists in Australia might benefit from the synergy between climate and land-use change.

Despite the potential for many birds of tropical savannas to track the geographic shift in their suitable climate space, the realization of this range shift may depend on whether land is available or has been anthropogenically modified to the extent of being unsuitable habitat (Pearson 2006). Many Australian tropical savanna bird species are predicted to show similar patterns to those documented elsewhere (Parmesan and Yohe 2003); tracing the movement of their suitable climate space across increasing latitudes. However, the projected future locations of greatest species richness—down the east coast and in far southwestern Australia—are heavily modified in comparison to the current savanna biome, with extensive urbanization and more intensive agriculture (Berry and Roderick 2006), a pattern predicted for tropical savanna birds in other parts of the world (Marini et al. 2009). The next important step in refining the understanding of the opportunities or constraints to fauna dispersing in response to changing climates will include other factors that limit or aid potential new distributions of species over time (Early and Sax 2011). For example, the location of conservation reserves in Australia and globally will need to be re-evaluated to assess their efficacy in light of the increasing evidence for species movements with shifting climate (Coetzee et al. 2009). Such re-evaluation may highlight the need for restoration of urban and agricultural areas to create suitable habitat to facilitate movements by range-shifting species (Shoo et al. 2011).

## Conclusions

Projected increases in extinction risk due to climate change have necessitated comprehensive climate change impact assessments across species assemblages (IPCC 2007b). The birds of Australian tropical savannas are projected to shift out of the arid zone as mean temperature increases, some into the southern savanna where rainfall is projected to increase, and others southward toward and along the east coast of Australia. Using realistic dispersal scenarios makes a substantial difference to the range projections when compared with no dispersal scenarios, and therefore appropriate dispersal scenarios are important for meaningful projections of species’ range-shifts. Overall, birds occurring in Australian tropical savannas are projected to decline in distribution size, and this response is reflected in assemblage measures such as species richness. While many species are predicted to change marginally, others species found in particular biogeographic zones (e.g., Cape York Peninsula and the arid zone), are predicted to show severe contraction and become increasingly vulnerable. Therefore an understanding of species dispersal capacities and the patchiness of available habitat in future destinations for these species is important in planning for the long-term persistence of species. Studies such as these support conservation adaptation programs by anticipating the effectiveness of current conservation for range-shifting species.

## Supporting information

**Fig. S1.** Mean annual temperature and annualprecipitation for current (1990), and the minimum, mean and maximumprojections for 2080.
